# Draft Genome Sequence of the Strict Anaerobe *Clostridium neopropionicum* X4 (DSM 3847^T^)

**DOI:** 10.1128/genomeA.00209-16

**Published:** 2016-04-14

**Authors:** Matthias H. Beck, Anja Poehlein, Frank R. Bengelsdorf, Bettina Schiel-Bengelsdorf, Rolf Daniel, Peter Dürre

**Affiliations:** a Institut für Mikrobiologie und Biotechnologie, Universität Ulm, Ulm, Germany; b Genomic and Applied Microbiology & Göttingen Genomics Laboratory, Georg-August University Göttingen, Göttingen, Germany

## Abstract

Here, we report the draft genome sequence of *Clostridium neopropionicum* X4 (DSM 3847^T^), a strictly anaerobic bacterium capable of fermenting ethanol and CO_2_ to propionate, acetate, and propanol. The genome consists of a single chromosome (3.19 Mb).

## GENOME ANNOUNCEMENT

*Clostridium neopropionicum* X4 was first isolated in 1982 from a mesophilic industrial anaerobic digester treating vegetable cannery wastewaters (1). Later, it was further characterized as an obligately anaerobic endospore-forming bacterium, being the first representative of propionate fermentation from ethanol with simultaneous acetate production (1, 2). Tholozan et al. (2) reported the formation of [2-^13^C]propionate, [1-^13^C]acetate, [2-^13^C]propanol, and traces of [3-^13^C]butyrate as products of [1-^13^C]ethanol fermentation, suggesting the absence of a randomizing pathway (2). The respective reactions of ethanol conversion to these metabolic end products are all exergonic (2). Meanwhile, *C. neopropionicum* was suggested to be assigned to the genus *Tyzzerella* (3).

The MasterPure complete DNA purification kit (Epicentre, Madison, WI, USA) was used to isolate chromosomal DNA. Illumina Nextera XT shotgun sequencing libraries were generated from the extracted DNA, and sequencing was performed with an Illumina MiSeq machine, as recommended by the manufacturer (Illumina, San Diego, CA, USA). Sequencing resulted in 2,669,816 300-bp paired-end reads. Reads were trimmed using Trimmomatic 0.35 (4) to remove sequences with quality scores <20 (Illumina 1.9 encoding) and adaptor sequences. The remaining 2,530,048 reads were used for the *de novo* assembly performed with the SPAdes genome assembler software version 3.6.2 (5). The assembly resulted in 29 contigs (>500 bp) and an average coverage of 182-fold. The genome of *C. neopropionicum* probably consists of a single circular chromosome (3.19 Mb), with an overall G+C content of 40.17%.

Automatic gene prediction was performed by using the software tool Prodigal (6). Genes coding for rRNA and tRNA were identified using RNAmmer (7) and tRNAscan (8), respectively. The Integrated Microbial Genomes-Expert Review (IMG-ER) system (9) was used for automatic annotation, which was subsequently manually curated by using the Swiss-Prot, TrEMBL, and InterPro databases (10).

The genome harbored 6 rRNA genes, 58 tRNA genes, 2,090 protein-coding genes with predicted functions, and 839 genes coding for hypothetical proteins. Genome analysis revealed the presence of several genes encoding alcohol dehydrogenases, including also an *adhE* gene (CLNEO_13930), which codes for a bifunctional aldehyde/alcohol dehydrogenase and a gene (CLNEO_02960) that putatively represents the NADP^+^-dependent ethanol dehydrogenase gene postulated by Tholozan et al. (2). Genes encoding key enzymes of the acrylate pathway (propionate-coenzyme A [CoA] transferase [Pct], lactoyl-CoA dehydratase [Lcd], and acrylyl-CoA reductase [Acr]) were also identified and show high sequence similarity to the corresponding genes described for *Clostridium propionicum*. These genes are organized in two clusters (*lcdCAB-pct* and *acrCBA*), which showed an arrangement identical to that in *C. propionicum* (11). Whole-genome comparison based on computed average nucleotide identity (ANI) revealed a similarity of >80% between *C. neopropionicum* and *C. propionicum*, which is clearly in contrast to earlier findings of only 18% DNA/DNA homology (2). However, our analysis and former 16S rRNA gene-based studies (12) suggest that *C. neopropionicum* and *C. propionicum* are highly related.

### Nucleotide sequence accession numbers.

This whole-genome shotgun project has been deposited at DDBJ/EMBL/GenBank under the accession no. LRVM00000000. The version described in this paper is version LRVM01000000.
